# Observation of the anomalous Nernst effect in altermagnetic candidate Mn_5_Si_3_

**DOI:** 10.1038/s41467-025-62331-7

**Published:** 2025-08-02

**Authors:** Antonín Badura, Warlley H. Campos, Venkata K. Bharadwaj, Ismaïla Kounta, Lisa Michez, Matthieu Petit, Javier Rial, Miina Leiviskä, Vincent Baltz, Filip Krizek, Dominik Kriegner, Jakub Železný, Jan Zemen, Sjoerd Telkamp, Sebastian Sailler, Michaela Lammel, Rodrigo Jaeschke-Ubiergo, Anna Birk Hellenes, Rafael González-Hernández, Jairo Sinova, Tomáš Jungwirth, Sebastian T. B. Goennenwein, Libor Šmejkal, Helena Reichlova

**Affiliations:** 1 Institute of Physics, Czech Academy of Sciences, Prague, Czechia; 2 Faculty of Mathematics and Physics, Charles University, Prague, Czechia; 3 Max Planck Institute for the Physics of Complex Systems, Dresden, Germany; 4 Institute of Physics, Johannes Gutenberg University Mainz, Mainz, Germany; 5 Aix Marseille University, CNRS, CINAM, AMUTECH, Marseille, France; 6 University. Grenoble Alpes, CNRS, CEA, Grenoble INP, IRIG-SPINTEC, Grenoble, France; 7 Faculty of Electrical Engineering, Czech Technical University in Prague, Prague 6, Czechia; 8 Solid State Physics Laboratory, ETH, Zürich, Switzerland; 9 Department of Physics, University of Konstanz, Konstanz, Germany; 10 Departamento de Física, Grupo de Investigación en Física Aplicada, Universidad del Norte, Barranquilla, Colombia; 11 Department of Physics, Texas A&M University, College Station, TX USA; 12 School of Physics and Astronomy, University of Nottingham, Nottingham, UK; 13 Max Planck Institute for Chemical Physics of Solids, Nöthnitzer Str. 40, 01187 Dresden, Germany

**Keywords:** Spintronics, Magnetic properties and materials

## Abstract

The anomalous Nernst effect generates a voltage transverse to an applied thermal gradient in some magnetically ordered systems. While the effect was considered excluded in compensated magnetic materials with collinear ordering, in the recently identified symmetry-class of altermagnets, the anomalous Nernst effect is possible despite the compensated collinear spin arrangement. In this work, we show that epitaxial Mn_5_Si_3_ thin films grown on Si manifest an anomalous Nernst effect with a finite spontaneous signal at zero magnetic field despite the vanishing spontaneous magnetization. We attribute this to the previously theoretically predicted and experimentally corroborated altermagnetism of epitaxial Mn_5_Si_3_ thin films grown on Si. The observed spontaneous anomalous Nernst coefficient reaches the value of 0.26 μV/K with the corresponding spontaneous Nernst conductivity of 0.22 A/(K  ⋅  m). To complement our measurements, we perform density-functional theory calculations of the momentum-resolved anomalous Nernst conductivity, highlighting the contributions of altermagnetic pseudonodal surfaces and ladder transitions to the Berry curvature. Our results illustrate the value of unconventional d-wave wave altermagnets composed of abundant and non-toxic light elements for thermo-electrics and spin-caloritronics.

## Abstract (web summary)

Altermagnets, unlike their conventional collinear antiferromagnetic counterparts, allow for an anomalous Nernst response despite their collinear compensated magnetic ordering. Here, Badura et al find such an anomalous Nernst effect at zero magnetic field in the altermagnetic candidate, Mn_5_Si_3_.

## Introduction

**Fig. 1:**
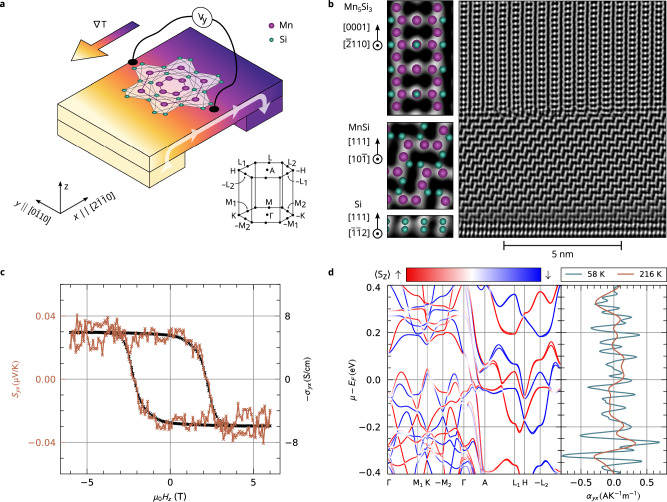
The anomalous Nernst effect in Mn_5_Si_3_. **a** Schematic illustration of the experiment: A longitudinal temperature gradient  ∇ *T* induces a spontaneous transverse voltage *V*_*y*_. **b** A cross-section of a typical sample, i.e., substrate/MnSi buffer layer/Mn_5_Si_3_ layer, captured by scanning transmission electron microscopy, including crystal structure models in the left panel. **c** Transverse Nernst signal *S*_*yx*_ as a function of applied magnetic field for a sample temperature of 216 K. The figure also includes the field dependence of the anomalous Hall conductivity for comparison. **d** Ab initio calculations of the band structure of Mn_5_Si_3_ (the left panel) including the calculated anomalous Nernst conductivities at 58 K and 216 K (the right panel).

The anomalous Nernst effect (ANE, see Fig. 1a) was traditionally considered only in ferromagnetic materials. However, recent studies have shown that it can also be observed in non-collinear antiferromagnets, such as ${{{{\rm{Mn}}}}}_{3}{{{\rm{Sn}}}}$^1^ and Mn_3_NiN^2^. In these materials, the ANE can arise from a finite Berry curvature in the momentum space and is allowed by the symmetry of the atomic positions and non-collinear spins of the frustrated magnetic lattice^3–6^. This leads to the presence of a sizable ANE despite the vanishing net magnetization. The potential to enhance the ANE response has renewed interest in this phenomenon both in ferromagnets^7,8^ and antiferromagnets^6,9^, with possibilities for its application in heat harvesting elements or heat flux sensors. Furthermore, the presence of ANE in a material enables the use of novel magnetic imaging microscopy techniques that rely on thermal gradients^10–13^. However, the complex spin arrangement of non-collinear antiferromagnets does not support long spin coherence, and the high ANE magnitude is often observed in non-collinear systems with heavy elements. In the broad family of compensated magnets with an unfrustrated collinear antiparallel ordering, the ANE was thought to be absent. This understanding should be revised because of the recently identified class of altermagnets, which combine collinear compensated magnetic order with anisotropic spin splitting in their electronic structure^14,15^. Epitaxial thin films of Mn_5_Si_3_ have recently been recognized as a candidate representative of this class^16,17^.

In the paramagnetic state, Mn_5_Si_3_ crystallizes in a hexagonal unit cell with the space group *P*6_3_/*mcm*. There are two non-equivalent sites of manganese atoms in its unit cell: four Mn atoms (Mn1) occupy the Wyckoff position 4d, while the remaining six are at the Wyckoff position 6g (Mn2)^18,19^. Upon the onset of magnetic order the behavior of bulk and thin films differ. While bulk Mn_5_Si_3_ undergoes two successive first-order transitions into antiferromagnetic phases, epitaxial strain causes an altermagnetic order in thin films^16,20–22^.

For bulk Mn_5_Si_3_, neutron-diffraction experiments^19,23,24^ establish that below 100 K, the crystal symmetry is reduced to orthorhombic accompanied by a collinear antiferromagnetic configuration with a symmetry combining time-reversal and half unit-cell translation, which forbids the ANE^25^. The second transition occurs at 60 K when the magnetic order becomes highly noncollinear and noncoplanar^24^. The presence of the ANE in this low-temperature phase is under discussion^25^.

Due to epitaxial strain, epitaxial Mn_5_Si_3_ thin films exhibit no structural transitions, as evidenced by X-ray diffraction measurements. Consequently, the unit cell remains hexagonal in the temperature range of 10–300 K^16,17^. This particular crystal structure in combination with a compensated collinear order between (90 ± 30) K and (240 ± 30) K^16,20–22^ leads to the breaking of translation and inversion symmetries while preserving a rotation symmetry connecting sublattices with opposite spins. These non-relativistic spin symmetries categorize the material as altermagnetic^15^. Although direct measurements of spin structure in thin films of compensated magnets are notoriously difficult, indirect experimental evidence supports the existence of the altermagnetic phase in these films by the measured anomalous Hall effect (AHE)^16,22^ despite a negligible net remanent magnetization. The proposed magnetic structure of Mn_5_Si_3_ films is consistent with the previous experimental studies^16,20,22^, including a recent work demonstrating that this structure is a key ingredient in explaining the characteristic anisotropy of angular-dependent magneto-transport^21^.

The AHE response in Mn_5_Si_3_ epilayers is spontaneous, i.e., it is sizable even in the absence of the magnetic field. It corroborates the altermagnetic mechanism of the time-reversal symmetry breaking in the electronic structure. The non-relativistic spin-split electronic structure is anisotropic in the momentum space with the d-wave symmetry^16^. Lowering the symmetry by the relativistic spin-orbit coupling then allows for a non-zero Berry curvature in the momentum space. Correspondingly, the AHE can be detected in measurements of the (0001)-oriented Mn_5_Si_3_ films, except for the case of the Néel vector aligned with the [0001]-axis for which the integral of the Berry curvature vanishes by symmetry, or for the Néel vector within the (0001)-plane, $(2\overline{1}\overline{1}0)$-plane or $(0\overline{1}10)$-plane for which the Hall vector, if allowed, is constrained by symmetry to the (0001)-plane of the thin film^16^.

In this combined experimental and theoretical work, we demonstrate that the spontaneous anomalous Nernst effect can also be observed in altermagnetic materials. Our calculations, in agreement with experimental results, reveal a spontaneous ANE in the altermagnetic candidate Mn_5_Si_3_ thin films, composed of light and earth-abundant elements. Our results suggest a new direction for observing and utilizing the ANE within the broad family of altermagnets, which also includes semiconductors that are favorable conduction types for thermoelectric.

## Results

For our experiments, we use 20 nm Mn_5_Si_3_(0001) films grown by molecular-beam epitaxy on intrinsic Si(111) substrates. Figure 1b shows a section of our Mn_5_Si_3_ epilayer along its [0001] direction as captured by scanning transmission electron microscopy (STEM). The STEM image displays the high quality of our film and its interface, as well as the 5-nm buffer layer of MnSi, which forms at the interface after annealing. The hexagonal crystal symmetry in magnetically ordered phase, obeying the altermagnetic symmetry requirements was confirmed by the X-ray diffraction measurements of lattice parameters^16^. Measurement of the temperature-dependent resistivity (see Supplementary Fig. S1a) shows characteristic features reported in Mn_5_Si_3_ epitaxial films^16^. Our samples exhibit the spontaneous AHE, as shown in Fig. 1c (black line). The spontaneous AHE is present in a wide temperature range as illustrated in Supplementary Figs. S1b and S1d with a characteristic feature below 120 K which was ascribed to the topological Hall effect arising from the non-collinear magnetic order^16,26^. See Methods and ref. ^17^ for more details about the layer growth and characterization.

**Fig. 2:**
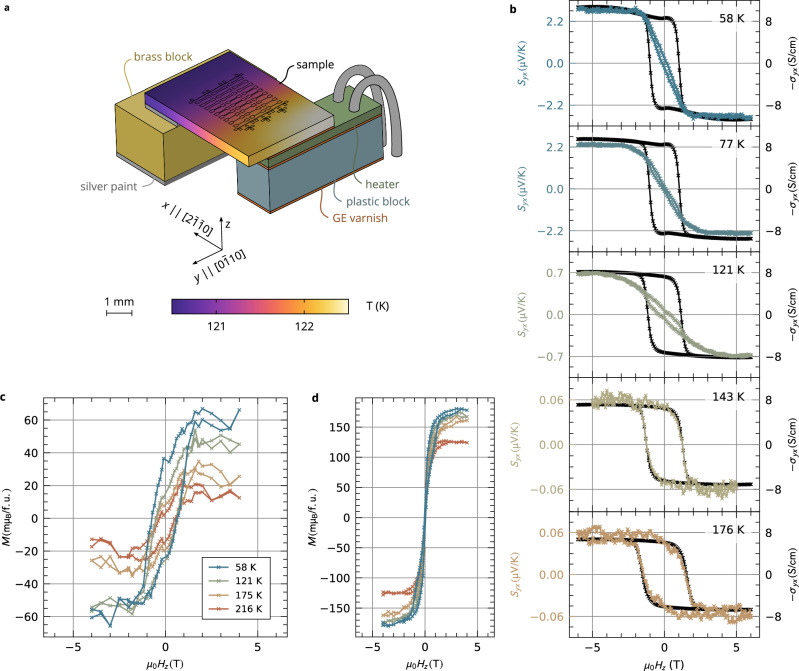
Temperature dependence of the anomalous Nernst effect in Mn_5_Si_3_. **a** Detailed image of the experimental setup: The longitudinal temperature gradient in the sample is induced by heating up the sample with a platinum resistive heater. The color map of the sample temperature for a particular setting was calculated by a finite-element simulation. **b** Magnetic-field dependence of the anomalous Nernst signal *S*_*yx*_ for five average sample temperatures. Each figure shows the anomalous Hall conductivity measured at the same temperature for comparison. **c** Dependence of the detected out-of-plane magnetization on the applied magnetic field for different sample temperatures. The figure features the spontaneous magnetization of our Mn_5_Si_3_ layers after subtracting a saturating contribution from the substrate. In the presented data, the dominating diamagnetic signal of the silicon substrate has been subtracted. **d** The raw data of magnetization in panel c, including a saturating contribution from the substrate.

To measure the ANE in our samples, we generate an in-plane temperature gradient along the $[\overline{2}110]$ direction by a macroscopic heater as illustrated in Fig. 2a: The sample is supported by a plastic block with a resistive heater on one side and by a brass block on the other side^27^. The different thermal conductivities of the plastic and brass blocks enhance the temperature gradient generated by the heater. To detect the thermovoltage generated in the Mn_5_Si_3_ layer, we lithographically defined Mn_5_Si_3_ strip structures, as shown in the detail of our design in Supplementary Fig. S2a. We detect the thermovoltage *V*_*y*_ transverse to the gradient direction as a function of the magnetic field applied along the z-axis (film’s c-direction) as illustrated in Fig. 1a. An example of such a field-dependent transverse thermopower *S*_*yx*_ is shown in Fig. 1c for a sample average temperature of 216 K. *S*_*yx*_ shows clear hysteretic behavior and saturation, a signature of the finite ANE in our samples. The saturation field and coercivity of *S*_*yx*_ match the field dependence of the transverse conductivity *σ*_*yx*_ shown in the same panel. The experimental manifestation of ANE is supported by our ab initio calculations of the anomalous Nernst conductivity *α*_*yx*_. The energy dependence of *α*_*yx*_ for temperature 58 K and 216 K is shown in Fig. 1d next to the spin-resolved electronic band structure of altermagnetic Mn_5_Si_3_. It reaches the value of 0.25 A/(K ⋅ m) at the Fermi energy for 58 K.

A crucial step in quantifying the Nernst coefficient *S*_*xy*_ is to determine the temperature gradient in the sample precisely. To do so, we measure the temperature at two places on the sample by deposited platinum strips. The precise spatial temperature distribution is simulated by the finite-element method in COMSOL MULTIPHYSICS^28^, where we used realistic geometry of our experimental setup and adjusted the surface heat transfer to fit the measured sample temperatures. The result is shown as a color map in Fig. 2a for the average sample temperature of 121 K. An example of the temperature gradient profile determined by the simulation is shown in Supplementary Fig. S2a: The temperature gradient is linear in the central patterned part of the sample and deviates from the linearity at the edges. The magnitude of the simulated thermal gradient along the *x*-axis varied between 0.04 K/mm at 58 K and 0.93 K/mm at 216 K.

Figure 2b presents *S*_*yx*_ measured at five different sample temperatures between 58 K and 176 K, complementing the 216 K measurement in Fig. 1c. We take into account only the odd-in-field part of the *S*_*yx*_ field dependence to remove the effect of the temperature gradient not being perfectly perpendicular to the lithographic bars. The figure also features the field dependence of the transverse conductivity *σ*_*yx*_ measured on the same sample at the same temperatures. Both *S*_*yx*_ and *σ*_*yx*_ are presented without the linear-in-field contribution of the ordinary Nernst effect and the ordinary Hall effect, respectively. The magnitudes of the saturated and zero-field (spontaneous) *S*_*yx*_ are summarized in Supplementary Fig. S4a. Both quantities are strongly temperature dependent: Saturated *S*_*yx*_ drops from  −(2.8 ± 1.1) μV/K at 58 K to  −(0.03 ± 0.01) μV/K at 216 K, and spontaneous *S*_*yx*_ changes from  −(0.26 ± 0.10) μV/K at 58 K to  −(0.03 ± 0.01) μV/K at 216 K. In contrast, the saturated *σ*_*yx*_ only slightly decreases from (10.6 ± 0.1) S/cm to (5.91 ± 0.02) S/cm. We also measured the field dependence of the Seebeck coefficient *S*_*xx*_. This allows us to determine the spontaneous Nernst conductivity *α*_*yx*_, which varies from ∣*α*_*yx*_∣ = (0.22 ± 0.11) A/(K ⋅ m) at 58 K to ∣*α*_*yx*_∣ = (0.04 ± 0.01) A/(K ⋅ m) at 216 K.

Our Mn_5_Si_3_ samples show a sizable ANE despite their small magnetization as illustrated in Fig. 2c: The figure shows the magnetic-field dependence of the out-of-plane component of the magnetization measured by SQUID magnetometry at different temperatures. The data corresponds to the spontaneous magnetization of our Mn_5_Si_3_ after subtracting the saturating contribution of the substrate and the sample holder. The raw data of the same measurements are given in Fig. 2d. We present the data without the dominating diamagnetic signal of the silicon substrate. The data show a clear hysteretic behavior with the saturation value of (45 ± 20) mμ_B_/f.u. at 58 K, which decreases to (10 ± 8) mμ_B_/f.u. at 216 K. The field dependence of the magnetization suggests that the Néel vector reversal is accompanied by small canting of the magnetic sublattice moments. We present an extended dataset of the magnetization curves in Extended Data Fig. 1c which highlights the vanishing hysteresis at 300 K. The observed spontaneous moment of (20 ± 10) mμ_B_/f.u. at 58 K is of the same order as the value 40 mμ_B_/f.u. obtained from our ab initio calculations.

**Fig. 3:**
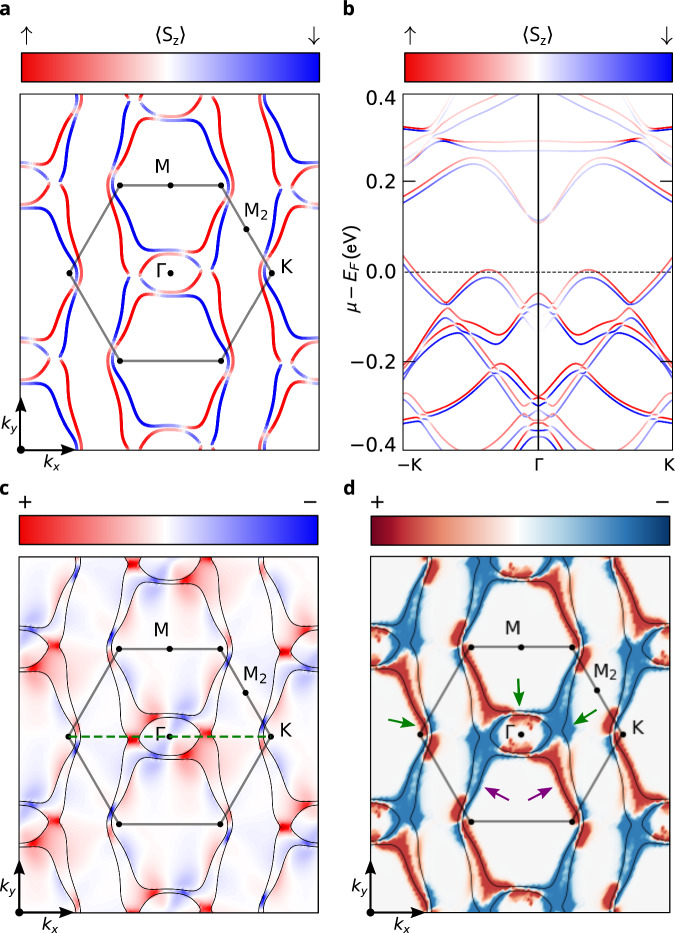
Relativistic Fermi surface with momentum-resolved Berry curvature and anomalous Nernst conductivity in the *k*_*z*_ = 0 plane. **a** Spin-resolved relativistic Fermi surface with gray solid lines outlining the first Brillouin zone. The color map shows the spin texture of the Fermi surface, with positive (negative) 〈*S*_*z*_〉 values shown in red (blue). **b** SOC-split electronic band structure along the high-symmetry path  − K − *Γ* − K. **c** *z*-component of the Berry curvature distribution with the color map showing positive (negative) values in red (blue). The black solid lines show the relativistic Fermi surface, and the green dotted line highlights the high-symmetry path used in (**b**). **d** Distribution of the momentum-resolved anomalous Nernst conductivity for the temperature 58 K, with positive (negative) values in red (blue). The green arrows show the contributions coming from the SOC-split pseudo-nodal surfaces and the purple arrows indicate contributions coming from the ladder transitions between altermagnetic-split energy bands.

To quantitatively understand the origin of the observed ANE, we performed first-principles calculations of the Nernst conductivity *α*_*yx*_. The electronic band structure (left panel in Fig. 1d) shows a large anisotropic spin-splitting, the hallmark of altermagnets, together with a spin-mixing induced by spin-orbit coupling. In Fig. 3a, we show the relativistic Fermi surface for the *k*_*z*_ = 0 plane (for the non-relativistic Fermi surface, see Supplementary Fig. S9a). The avoided band crossings along the *Γ* − K and *Γ* − M paths are induced by spin-orbit coupling (SOC) as illustrated in Fig. 3b for  − K − *Γ* − K, showing the momentum-dependent band spin-splitting.

We have studied the momentum dependence of the Berry curvature and momentum-resolved anomalous Nernst conductivity (ANC) in altermagnetic Mn_5_Si_3_ from first principles (see Methods). We have identified that the leading characteristic contributions to the effects can be best illustrated on the *k*_*z*_ = 0 plane shown in Fig. 3a, c, d. Contributions to the Berry curvature were analyzed in an earlier theoretical study of the d-wave altermagnetic phase of RuO_2_. Prominent contributions were found to originate from Weyl points at crossings between bands of the same spin, pseudonodal surfaces whose spin degeneracy is protected by the altermagnetic spin symmetries in the absence of SOC and is lifted by SOC, and ladder transitions from regions where a very small gap between spin-up and spin-down bands exists without SOC^29^. We emphasize that the presence of spin-flip pseudonodal surfaces distinguishes altermagnets from other magnetic systems, such as ferromagnets and non-collinear antiferromagnetic. As illustrated in Fig. 3c, our calculations show that the main contribution to the Berry curvature in altermagnetic Mn_5_Si_3_ comes from the pseudonodal surfaces and ladder transitions between altermagnetic split bands. (Note that Weyl points are found to be absent near the Fermi energy in altermagnetic Mn_5_Si_3_). In Fig. 3d, we show the corresponding momentum-resolved ANC for the temperature of 58 K with highlighted pseudonodal surfaces (green arrows) and ladder transitions (purple arrows). The ANC-momentum-space distributions calculated at 121 K, 176 K, and 216 K are shown in Supplementary Fig. S9b–d. The reciprocal space dependence of the ANC is computed via Eq. (4), without performing the integration over the Brillouin zone (in Eq. (3)).

In the right panel of Fig. 1d, we show the integrated anomalous Nernst conductivity *α*_*yx*_ values for different temperatures as a function of the chemical potential. The Nernst conductivity calculations yield 0.25 A/(K ⋅ m) for 58 K and 0.12 A/(K ⋅ m) for 216 K. The temperature dependencies of calculated and measured *α*_*yx*_ are compared in the Supplementary Fig. S4b. Both dependencies decrease with increasing temperature. The low-temperature experimental values agree with the calculated ones within the indicated uncertainty. The high-temperature values differ by a factor of two. This discrepancy might be caused by the slight shift of the expected position of the Fermi energy (see Supplementary Fig. S8) or by the possible phononic contributions which were not taken into account in the calculations.

*S*_*yx*_ shows a complex field-dependence compared to *σ*_*yx*_ as illustrated in Fig. 2b: The anomalous Nernst and Hall dependencies are nearly identical above 140 K, while the low-temperature *S*_*yx*_ have pronounced sigmoid character. This leads us to the hypothesis that the *S*_*yx*_ signal is composed of at least two competing contributions with different temperature dependencies. To disentangle them, we analytically describe the low-temperature sigmoid character of the *S*_*yx*_ curves by an error function. When the sigmoid contribution is subtracted, only a spontaneous part of *S*_*yx*_ prevails with the identical field dependence as *σ*_*yx*_. The analysis is robust in the whole temperature range, always giving spontaneous *S*_*yx*_ proportional to *σ*_*yx*_. This analysis strategy is illustrated in Supplementary Fig. S3. The sigmoid contribution vanishes above 170 K, as quantitatively shown in Supplementary Fig. S4a. We attribute the spontaneous component of *S*_*yx*_ to be a direct consequence of the altermagnetic spin-split electronic structure of our Mn_5_Si_3_ epilayers, analogous to our previous reports of the AHE in the material^16^. The interpretation of the sigmoid contribution is less clear. It cannot be attributed to the Brillouin magnetism which would not be expected up to 100 K. Because it is present only in the lower temperature range it may be related to the complex low-temperature magnetic phase of Mn_5_Si_3_^19,23,24^ where the topological Hall effect was reported^16,26^. However, a systematic study of the low-temperature phase of Mn_5_Si_3_^30^ including the sigmoid contribution to the ANE and comparing the low-temperature thin-film and bulk phases^25^ is an important future task.

**Fig. 4:**
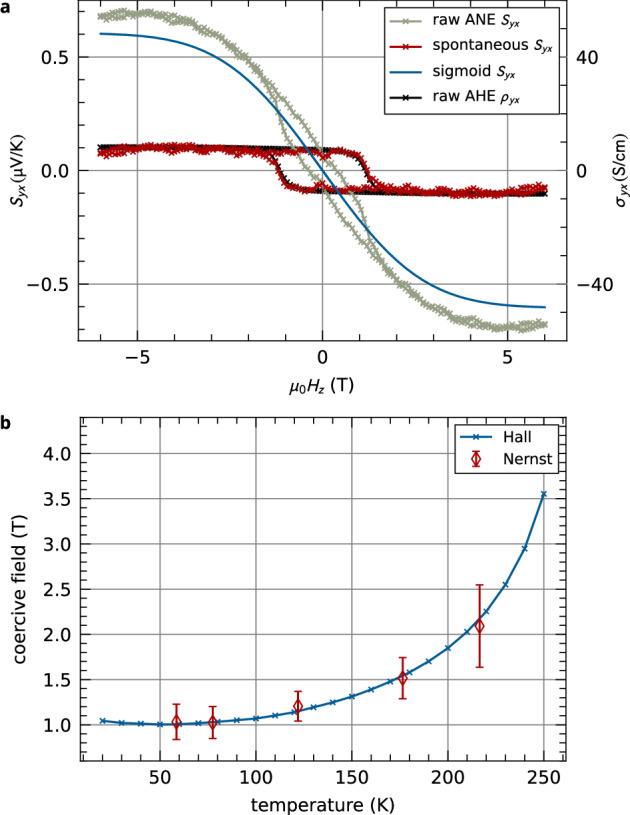
Decomposition of the anomalous Nernst signal. **a** The anomalous Nernst signal measured at 121 K decomposed into its spontaneous and sigmoid contributions. The anomalous Hall signal is also plotted for comparison. **b** Temperature dependence of the coercive field of the spontaneous ANE contribution together with the respective dependence for the AHE.

The separation of the spontaneous and sigmoid *S*_*yx*_ contributions enables us to quantify the temperature dependence of the *S*_*yx*_ coercive field and compare it with *σ*_*yx*_ as shown in Fig. 4b. Both signals show the same increasing temperature dependence of coercivity. In a ferromagnet, the common expectation is that the coercive (reorientation) field decreases with increasing temperature, usually because of the decreasing magnetocrystalline anisotropy^31,32^. The situation in compensated magnetic materials is, however, more complex: for example, the observed increase of the reorientation (spin-flop) field in collinear MnF_2_ was ascribed to its temperature-dependent and anisotropic response to the applied magnetic field^33^. In our Mn_5_Si_3_ thin films, however, we do not observe any reorientation transition in the reachable magnetic field (see Fig. 2c). Instead, we expect that the hysteresis in the Néel vector reversal originates from the competition of the magnetocrystalline anisotropy and the Dzyaloshinskii-Moriya interaction. These effects have generally different temperature dependencies, which can result in an increasing coercivity with temperature. The temperature dependence of the magnetocrystalline anisotropy may be affected by the complex temperature dependence of the lattice parameters^16^.

## Discussion

**Fig. 5:**
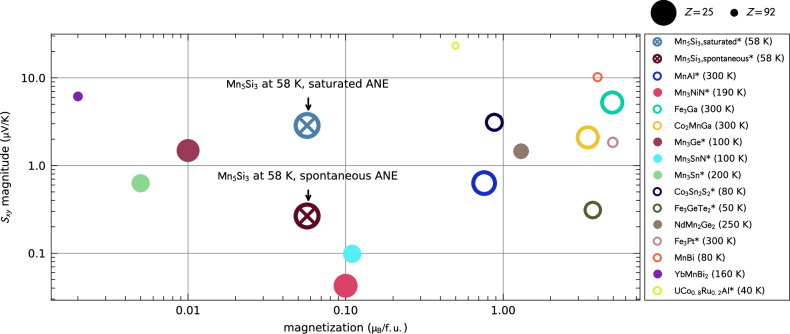
Comparison of the anomalous Nernst effect measured in epitaxial Mn_5_Si_3_ and in other materials^1,2,7,9,27,54–62^. The ANE magnitude *S*_*xy*_ is captured as a function of materials’ magnetization. The symbol size linearly decreases with the highest atomic number of materials’ formula. Antiferromagnetic materials are indicated by full circles, while ferromagnets are denoted by hollow circles and the altermagnet by a crossed circle. Materials showing the spontaneous Nernst effect are labeled by an asterisk.

We have demonstrated a significant and spontaneous ANE in an altermagnetic candidate composed of light elements. Fig. 5 presents a comparison of the ANE coefficients observed in various magnetic compounds depending on their magnetization per formula unit, including ferromagnets (hollow circles), non-collinear antiferromagnets (full circles), and our altermagnetic Mn_5_Si_3_ (crossed circle). Materials showing spontaneous ANE are labeled by an asterisk. Our material showcases ANE values comparable to those of other materials, despite having vanishing magnetization, collinear magnetic order, and being constituted of light elements.

Unlike ferromagnets or non-collinear antiferromagnets, altermagnets are characterized by the presence of symmetry pseudonodal surfaces in their electronic bands. This unique feature can contribute to the Berry curvature and enhance the ANC as demonstrated by our calculations in Mn_5_Si_3_.

The maximum anomalous Nernst conductivity value ∣*α*_*yx*_∣ = (0.22 ± 0.11) A/(K ⋅ m) we determine for Mn_5_Si_3_ at 58 K is comparable to *α*_*yx*_ reported for noncollinear antiferromagnets, such as ${{{{\rm{Mn}}}}}_{3}\,{{{\rm{Sn}}}}$ with 0.30 A/(K ⋅ m) at 150 K^1^, though it is smaller than the values for YMnBi_2_ (10 A/(K ⋅ m) at 75 K)^9^ and Co_2_MnGa with 2.0 A/(K ⋅ m) at 300 K^34^.

Unlike non-collinear magnets, the collinear spin arrangement of altermagnets allows for the generation of spin currents with well-defined spin-quantization axis or long spin coherence^35^. Consequently, altermagnets are highly advantageous for multi-functional spintronic and spincaloritronics devices. In such devices, a single layer can, for instance, enable both the coherent generation or propagation of spin currents and the efficient spontaneous ANE.

Although we observe finite but small saturation magnetization of 45 mμ_B_/f.u., let us emphasize that it is not the prime source of AHE and ANE as shown by the first-principles calculations. Instead, the AHE and ANE are dominated by the strong altermagnetic breaking of the time-reversal symmetry in the electronic structure in the absence of a net magnetization, combined with the relativistic spin-orbit coupling. An absence of a correlation between the atomic mass and the ANE is highlighted in Fig. 5 where the symbols’ size linearly decreases with the highest atomic number of the compound (the largest symbols represent the lightest elements). This observation highlights the importance of not limiting the search for high ANE performance materials solely to heavy elements. Additionally, we note that, unlike many materials listed in Fig. 5, our Mn_5_Si_3_ is in thin film form, which often reduces the ANE magnitude. Therefore, we anticipate that higher ANE values could be discovered in altermagnetic bulks.

In summary, we provide experimental evidence that altermagnetic materials can host a spontaneous ANE despite their collinear magnetic order and vanishing net magnetization, and without the need of heavy elements. We demonstrate a sizable anomalous Nernst coefficient in the altermagnetic candidate Mn_5_Si_3_ in the form of epitaxial thin layers. At 58 K, the effect shows spontaneous *S*_*yx*_ = −(0.26 ± 0.10) μV/K, saturated *S*_*yx*_ = −(2.8 ± 1.1) μV/K, and spontaneous ∣*α*_*yx*_∣ = (0.22 ± 0.11) A/(K ⋅ m). Both the spontaneous and saturated *S*_*yx*_ drop to *S*_*yx*_ = −(0.03 ± 0.01) μV/K at 216 K with the spontaneous ∣*α*_*yx*_∣ = (0.04 ± 0.01) A/(K ⋅ m). The Nernst effect measurements are supported by our first-principles calculations of the Mn_5_Si_3_ electronic band structure and the Nernst conductivity, which yield *α*_*yx*_ = 0.25 A/(K ⋅ m) at 58 K. Our momentum-resolved ANC calculation in Mn_5_Si_3_ reveals contributions from altermagnetic pseudonodal surfaces and ladder transitions to the Berry curvature, while suggesting the absence of Weyl points near the Fermi level.

By demonstrating the ANE in an altermagnet, we introduce a new category of compensated magnetic materials for exploring the ANE. This expands the field beyond the more complex non-collinear magnetic structures or materials with heavy elements, marking a significant broadening of the scope for the ANE research. The search for materials with high and robust ANE magnitude is one of the key challenges for fully exploiting their application potential. Furthermore, it will be important to explore the universal validity of the Mott rule in this class of materials. Although the ANE and the AHE typically share the same symmetry, the Mott rule is not a fundamental principle and can be violated^36^. Moreover, in materials where the AHE and the ANE have the common Berry-curvature origin, the ANE can exhibit some features distinct from the AHE^37^ and its magnitude can be influenced by various means including spin fluctuations at transition temperature^38^.

## Methods

### Sample preparation

The Mn_5_Si_3_ thin films were grown by molecular beam epitaxy (MBE) in an ultra-high vacuum system with a base pressure better than 2 × 10^−7^ Pa. Elemental Mn and Si were flux matched in a 5: 3 ratio using a quartz microbalance and co-deposited on the intrinsic Si(111) substrate (*R* > 10,000 *Ω* ⋅ cm) heated to 170 °C. The films were subsequently annealed up to  ≈300 °C, the temperature for which a clear $(\sqrt{3}\times \sqrt{3})R\,3{0}^{\circ }$ reconstruction is observed on the RHEED pattern. Ex situ X-ray diffraction (XRD) using a high brilliance rotating anode showed a good crystalline quality of the Mn_5_Si_3_(0001) thin films whose growth was promoted by the formation of a thin MnSi(111). A more detailed description of the growth process can be found in ref. ^17^.

For the measurement of the anomalous Nernst effect, we used optical lithography and argon ion beam etching to pattern our Mn_5_Si_3_ layers into two distinct lithographic designs illustrated in the Supplementary Fig. S2. The platinum thermometers of  ≈50 nm thickness were deposited by magnetron sputtering.

### Magnetometry measurements

Magnetic characterization of our Mn_5_Si_3_ thin films was carried out using a SQUID MPMS XL 5.0 magnetometer. The as-grown sample was cleaned with acetone, alcohol, and DI water, before measurement and mounted with two dots of glue (X60) on a sample holder made of two quartz capillaries. Field-dependent magnetization was measured at different temperatures for magnetic field strengths ranging from  ±4 T applied along the film normal. The signal is dominated by the diamagnetism of the silicon substrate, which accounts for 96% of the signal at 4 T. After eliminating the linear-in-field diamagnetic contribution, the magnetic response contains two components: a non-hysteretic part linked to the substrate and sample holder and a hysteretic part that we attribute to the Mn_5_Si_3_ film, due to a canting of the moments of the two sublattices.

### High-resolution scanning transmission electron microscopy imaging and lamellae preparation

The cross-sectional lamellae of selected samples were prepared by Focused Ion Beam (FIB) (Helios 5 UX from Thermo Fisher Scientific) using AutoTEM 5 software (Thermo Scientific, the Netherlands) at ScopeM, ETH Zurich. A protective carbon layer was deposited on the selected region of interest first by an electron beam (2 KV, 13 nA) and subsequently by an ion beam (30 kV, 1.2 nA). The chuck milling and lamellae thinning were done at 30 kV with FIB current from 9 nA to 90 pA. Finally, the lamellae were polished at 5 KV (17 pA) and finished at 2 kV (12 pA). The expected thickness was below 50 nm. The samples were kept in vacuum between the FIB and TEM measurements (except for loading and unloading).

The STEM images were acquired with a JEOL ARM200F cold field emission gun scanning transmission electron microscope located at the Binnig and Rohrer Nanotechnology Center Noise-free laboratories at IBM Research Europe. The images were acquired at 200 kV and were further processed using FIJI - Fiji is just ImageJ freeware^39^. The FFT pattern of the images was masked by a circular aperture. After masking, a constant threshold was subtracted before performing an inverse FFT of the spectra to remove the background between the high-intensity spots corresponding to Bragg peaks. In selected images, the contrast was normalized by the contrast normalization plugin for better visualization of lighter elements.

### Density-functional theory calculations

We calculated the electronic structure of Mn_5_Si_3_ (space group *P*6_3_/*mcm*, No. 193) in the pseudo-potential DFT code Vienna Ab initio Simulation Package (VASP)^40^ within Perdew-Burke-Ernzerhof (PBE) + SOC generalized gradient approximation (GGA)^41–43^. The lattice constants for the hexagonal unit cell containing two formula units (f.u.) are *a* = *b* = 6.901 Å and *c* = 4.795 Å – consistent with the experimental values reported in refs. ^16,17^. We switched off symmetrization and converged the self-consistent calculations on a 9 × 9 × 12 k-point grid using an energy cutoff of 520 eV, energy convergence criterion of 0.5 × 10^−8^ eV and the tetrahedron smearing method with Blöchl corrections and smearing parameter 0.1 eV. The converged ground state has Fermi energy 9.945 eV and a total magnetization of 40 mμ_*B*_/f.u., where μ_*B*_ is the Bohr magneton. We plotted the spin-resolved electronic band structure (left panel in Fig. 1d) using the PyProcar Python library for electronic structure pre/post-processing^44^.

The Néel vector was defined along the direction [111] and remains virtually unchanged after the self-consistent electronic minimization. The magnetic Mn atoms have magnetic moments of  ≈2.52 μ_B_ along the direction of the Néel vector. The net magnetization is  ≈0.0409 μ_B_/f.u. in the direction defined in the spherical coordinate system by *θ* = 121° and *ϕ* = 244°. The magnitude of the net magnetization is then  ≈1.6% of the Mn atoms.

To calculate the anomalous Nernst conductivity (ANC; right panel in Fig. 1d) from our DFT results, we initially obtained the Wannier functions of Mn_5_Si_3_ using the Wannier90 code^45^. Then, we incorporated these Wannier functions on the WannierBerri python library^46^ using a 26 × 26 × 30 k-point grid and a Fermi-Dirac smoother with smearing parameter 0.86 meV to evaluate the quantities reliant on the Berry curvature^47^

1 $$\begin{array}{l}{\Omega }_{yx}^{n}({{{\bf{k}}}})=-i\left[\frac{\partial }{\partial {k}_{x}}\left\langle {u}_{n{{{\bf{k}}}}}\right| \frac{\partial }{\partial {k}_{y}}\left| {u}_{n{{{\bf{k}}}}}\right\rangle \right.\\ \left.-\frac{\partial }{\partial {k}_{y}}\left\langle {u}_{n{{{\bf{k}}}}}\right| \frac{\partial }{\partial {k}_{x}}\left| {u}_{n{{{\bf{k}}}}}\right\rangle \right],\end{array}$$

where **k** = (*k*_*x*_, *k*_*y*_, *k*_*z*_) is the crystal momentum and $\left| {u}_{n{{{\bf{k}}}}}\right\rangle$ is the periodic part of the Bloch state with band index *n*. Finally, we obtained the ANC for temperature *T* = 1 K via the Mott relation valid in the low-temperature limit^48^

2 $${\alpha }_{yx}(\mu )\approx \frac{{\pi }^{2}}{3}\frac{{k}_{B}^{2}T}{e}{\sum}_{n}{\int}_{BZ}\frac{d{{{\bf{k}}}}}{{(2\pi )}^{3}}{\Omega }_{yx}^{n}({{{\bf{k}}}})\frac{\partial f({\varepsilon }_{n{{{\bf{k}}}}},\mu )}{\partial {\varepsilon }_{n{{{\bf{k}}}}}},$$

where *e* is the electron elementary charge, *μ* is the chemical potential, *ε*_*n***k**_ are the energy eigenvalues and $f(\varepsilon,\mu )={\left\{1+\exp \left[\left(\varepsilon -\mu \right)/{k}_{B}T\right]\right\}}^{-1}$ is the Fermi-Dirac distribution with *k*_*B*_ the Boltzmann constant. The integration runs over the whole Brillouin zone (BZ).

For the ANC at higher temperatures, we first obtained the anomalous Hall conductivity (AHC) at *T* = 0 K^47^

3 $${\sigma }_{yx}^{T=0}(\varepsilon )=-\frac{{e}^{2}}{\hslash }{\sum}_{n}{\int}_{BZ}\frac{d{{{\bf{k}}}}}{{(2\pi )}^{3}}{\Omega }_{yx}^{n}({{{\bf{k}}}})\Theta (\varepsilon -{\varepsilon }_{n{{{\bf{k}}}}}),$$

where *Θ*(*x*) is the Heaviside function. Then, we used our AHC results - consistent with the calculations previously reported in ref. ^16^ - to obtain the ANC via the Mott relation^29,48–51^

4 $${\alpha }_{yx}(\mu )=-\frac{1}{e}\int\,d\varepsilon \frac{\partial f(\varepsilon,\mu )}{\partial \mu }{\sigma }_{yx}^{T=0}(\varepsilon )\frac{\varepsilon -\mu }{T}.$$

At low temperatures, Eq. (4) reduces to ${\alpha }_{yx}\approx \frac{{\pi }^{2}}{3}\frac{{k}_{B}^{2}T}{e}\frac{d{\sigma }_{yx}}{d\varepsilon }$, which we used to verify our results for *T* = 1 K obtained from Eq. (2).

We test our Nernst conductivity calculations on iron. For iron at 300 K, we obtain *α*_*xy*_ = −0.31 A/(K ⋅ m) which is in very good agreement with the experimental observation of *α*_*xy*_ ≈ −0.5 A/(K ⋅ m)^52^.

### Magnetotransport measurements

The transport experiments were conducted in an Oxford Instruments Integra AC cryostat equipped with a 3D superconducting magnet. The temperature gradient in the sample was generated by an external heater as illustrated in Fig. 2a and photographed in Supplementary Fig. S2d. As a heater, we used the platinum resistor Pt2000. The applied heating power ranged from 0.2 to 2 W. The sample temperature was detected by two on-chip platinum strips located on the opposite ends of the sample with respect to the applied in-plane temperature gradient. We calibrated the resistance of the platinum thermometers by a slow cool-down of the sample.

The transverse Nernst voltage was detected by Keithley Nanovoltmeters 2182A. The Nernst coefficient *S*_*yx*_ was determined from the measured voltage *V*_*y*_ and the simulated temperature gradient *x*-component (∇*T*)_*x*_ as: *S*_*yx*_ = *E*_*y*_/(∇*T*)_*x*_ = *V*_*y*_/(*l* ⋅ (∇*T*)_*x*_) where *l* is the length of the transverse Mn_5_Si_3_ contact. The main contribution to the error of the *S*_*yx*_ is the determination of the temperature gradient, which we determine with the absolute error of 0.02 K/mm. As shown in Supplementary Fig. S5 for 121 K, we evaluated *S*_*yx*_ at multiple devices on the chip with different widths of the contact. For each device, we used the value of the (∇*T*)_*x*_ determined from the finite-element simulations as illustrated in Supplementary Fig. S2a for 121 K. The mean value of saturated *S*_*yx*_ at 121 K for the three presented devices is  −0.646 μV/K with the standard deviation of 0.067 μV/K which agrees with the value presented in Fig. 2b and Supplementary Fig. S4a of (−0.679 ± 0.097) μV/K.

To evaluate the magnetic field dependence of *S*_*yx*_, we treat the data as illustrated in Supplementary Fig. S3a: We only consider its odd-in-field component to eliminate the effect of the temperature gradient not being perfectly perpendicular to the lithographic bars. We suppose that the strong even-in-field contribution corresponds to the magneto-Seebeck effect arising due to the misalignment of contact bonds. Furthermore, we subtract the linear-in-field contribution of the ordinary Nernst effect. The slope of the ordinary Nernst effect reaches  −2.44 μV/(K ⋅ T) at 58 K and increases to  −0.12 μV/(K ⋅ T) at 216 K. For the further analysis of anomalous *S*_*yx*_, we separate the signal into its spontaneous and sigmoid contributions. We describe the sigmoid contribution by an error function *f*(*μ*_0_*H*_*z*_):

5 $$f({\mu }_{0}{H}_{z})=\frac{2\,A}{\pi }\int_{0}^{{\mu }_{0}{H}_{z}}{e}^{-\frac{{t}^{2}}{B}}{{{\rm{d}}}}t.$$

where *A* and *B* are parameters, describing the magnitude and saturation field of the sigmoid contribution, respectively. Note that our choice of the error function over other sigmoid functions (e.g., hyperbolic tangent) is arbitrary. The spontaneous contribution to *S*_*xy*_ is the difference between anomalous *S*_*xy*_ and *f*(*μ*_0_*H*_*z*_).

We determined the magnetic field dependence of the Nernst conductivity *α*_*yx*_ via the relation

6 $${\alpha }_{yx}={\sigma }_{xx}\cdot {S}_{yx}+{\sigma }_{yx}\cdot {S}_{xx},$$

where *S*_*xx*_ is the Seebeck coefficient and *σ*_*xx*_ is longitudinal conductivity^51^. In Eq. (6), we use the *S*_*yx*_ and *σ*_*yx*_ values from Fig. 2b, i.e., odd-in-field component of raw transverse thermoelectric signal and conductivity without the linear-in-field contributions, which is not influenced by the buffer layer or the substrate. The values of *S*_*xx*_ and *σ*_*xx*_ were measured at a separate sample with a lithographically patterned Hall bar (see Supplementary Fig. S2b). *S*_*xx*_ was determined in the same experimental geometry as *S*_*xy*_, i.e., in the elevated configuration illustrated in Fig. 2a, via the formula *S*_*xx*_ = *E*_*x*_/(∇*T*)_*x*_ = *V*_*x*_/(*d* ⋅ (∇*T*)_*x*_) (*d* is the length of the longitudinal contact). The measured field-dependencies of *S*_*xx*_ and *σ*_*xx*_ are shown in Supplementary Fig. S6a. We present the *α*_*yx*_ field dependencies at different temperatures in Supplementary Fig. S6b. The values of spontaneous *α*_*yx*_ correspond to *α*_*yx*_ at zero magnetic field.

When determining *α*_*yx*_ from Eq. (6), the resulting value may be influenced by the MnSi buffer and the substrate contributions to the measured *S*_*xx*_ and *σ*_*xx*_. We conducted a dedicated finite-element simulation to describe the *S*_*xx*_ signal generated by a Mn_5_Si_3_/MnSi stack on a silicon substrate. The left panel of the Supplementary Fig. S7 shows a distribution of the temperature gradient in such a stack simulated by COMSOL when keeping the opposite sides of the sample at 214 K and 216 K, respectively. The figure shows that the silicon substrate fully dictates the temperature distribution in the device despite the different thermal conductivities of Mn_5_Si_3_ and MnSi compared to silicon. The right panel presents the distribution of the Seebeck signal, showing that the large Seebeck coefficient of the substrate does not influence the signal detected at the device. This is due to the very small electric conductivity of the silicon substrate. The Seebeck signal at the device is homogeneous, and slightly influenced by the presence of the MnSi buffer — the difference to the pure Mn_5_Si_3_ value is approx. 26%. As a consequence, we use the as-measured *S*_*xx*_ values in Eq. (6) and include this error of *S*_*xx*_ to the error bar of *α*_*yx*_. The finite-element simulations at other temperatures show the same trend keeping the error below 26%. Since the electrical conductivity of the MnSi is similar to Mn_5_Si_3_ (e.g., 6500 S/cm for Mn_5_Si_3_ and 4500 S/cm for MnSi at 216 K) and due to the small thickness of the MnSi buffer layer, we use the as-measured values of *σ*_*xx*_ to calculate *α*_*yx*_.

### Finite-element simulations of temperature distribution

We simulated the distribution of the temperature in our sample by the finite-element method (FEM) in COMSOL MULTIPHYSICS^28^. We used realistic simulation geometry illustrated in Fig. 2a, which precisely reflects our experimental setup, including the chip carrier, the acrylonitrile styrene acrylate (ASA) and brass blocks, the platinum heater (in an alumina ceramic package), as well as the sample itself. The bonding wires were also taken into account. The simulation was performed using material-specific physical parameters as illustrated in Supplementary Table S1 showing the used thermal conductivity and heat capacity values. For certain materials, we used temperature-dependent material properties.

Our FEM model includes the heat equation with the mixed boundary condition of the constant temperature at the interfaces in contact with the chip carrier and constant heat flux elsewhere. The heat transfer coefficient is a fitting parameter of our model that we adjust to reach the temperatures experimentally observed on the on-chip platinum thermometers. The coefficient is temperature-dependent and increases from 26 W/(m^2^ ⋅ K) at 58 K to 89 W/(m^2^ ⋅ K) at 216 K. The sample heating is simulated via the Joule heating by the charge current in the platinum heater. The numerical mesh used in the simulation is visualized in the Supplementary Fig. S2c.

As illustrated in the Supplementary Fig. S2a, the simulated temperature gradient is linear in the measurement area of the sample, where it reaches  −∂*T*/∂*x* = 0.04 K/mm for the sample temperature of 58 K, 0.34 K/mm for 121 K, 0.74 K/mm for 176 K, 0.93 K/mm 216 K. Outside the measurement area, the temperature gradient is not monotonous and gains a finite out-of-plane component.

## Supplementary information

Supplementary Information

Peer Review File

## Data Availability

All data supporting the findings of this study are included within the main text or Supplementary Information. Any additional requests for information can be directed to the corresponding author. Source data for figures in the main text are available via Zenodo data repository^53^.

## Code availability

In the manuscript, we used the Vienna Ab initio Simulation Package (VASP) for our pseudo-potential DFT calculations. The package can purchased from https://www.vasp.at/. We also used the Wannier90 code available at https://wannier.org/ and the WannierBerri python library (accessible in the repository https://github.com/wannier-berri). All input files, post-processing procedures and scripts used to evaluate experimental and theoretical data are available from the authors on request.
