# Supplementary material for: Observation of the anomalous Nernst effect in altermagnetic candidate Mn5Si3
Source: Nat Commun. 2025 Aug 2;16:7111. doi: 10.1038/s41467-025-62331-7 (PMC12318009; doi:10.1038/s41467-025-62331-7)
Supplement: Supplementary file 1 — Supplementary Information [file 41467_2025_62331_MOESM1_ESM.pdf]

Supplementary Information: Observation of the anomalous Nernst effect in altermagnetic candidate Mn_5Si_3

Antonín Badura,[1,2,*] Warlley H. Campos,[3,4] Venkata K. Bharadwaj,[4] Ismaïla Kounta,[5] Lisa Michez,[5] Matthieu Petit,[5] Javier Rial,[6] Miina Leiviskä,[1] Vincent Baltz,[6] Filip Krizek,[1] Dominik Kriegner,[1] Jakub Železný,[1] Jan Zemen,[7] Sjoerd Telkamp,[8] Sebastian Sailler,[9] Michaela Lammel,[9] Rodrigo Jaeschke-Ubiergo,[4] Anna Birk Hellenes,[4] Rafael González-Hernández,[10,4] Jairo Sinova,[4,11] Tomáš Jungwirth,[1,12] Sebastian T. B. Goennenwein,[9] Libor Šmejkal,[4,1,3] and Helena Reichlova[1]

[1] *Institute of Physics, Czech Academy of Sciences, Prague, Czechia*

[2] *Faculty of Mathematics and Physics, Charles University, Prague, Czechia*

[3] *Max Planck Institute for the Physics of Complex Systems, Nöthnitzer Str. 38, 01187 Dresden, Germany*

[4] *Institute of Physics, Johannes Gutenberg University Mainz, Mainz, Germany*

[5] *Aix Marseille Univ, CNRS, CINAM, AMUTECH, Marseille, France*

[6] *Univ. Grenoble Alpes, CNRS, CEA, Grenoble INP, IRIG-SPINTEC, Grenoble, France*

[7] *Faculty of Electrical Engineering, Czech Technical University in Prague, Technická 2, 160 00, Prague 6, Czechia*

[8] *Solid State Physics Laboratory, ETH, Zürich, Switzerland*

[9] *Department of Physics, University of Konstanz, Konstanz, Germany*

[10] *Grupo de Investigación en Física Aplicada, Departamento de Física, Universidad del Norte, Barranquilla, Colombia*

[11] *Department of Physics, Texas A&M University, College Station, Texas, USA*

[12] *School of Physics and Astronomy, University of Nottingham, Nottingham, United Kingdom*

* badura@fzu.cz

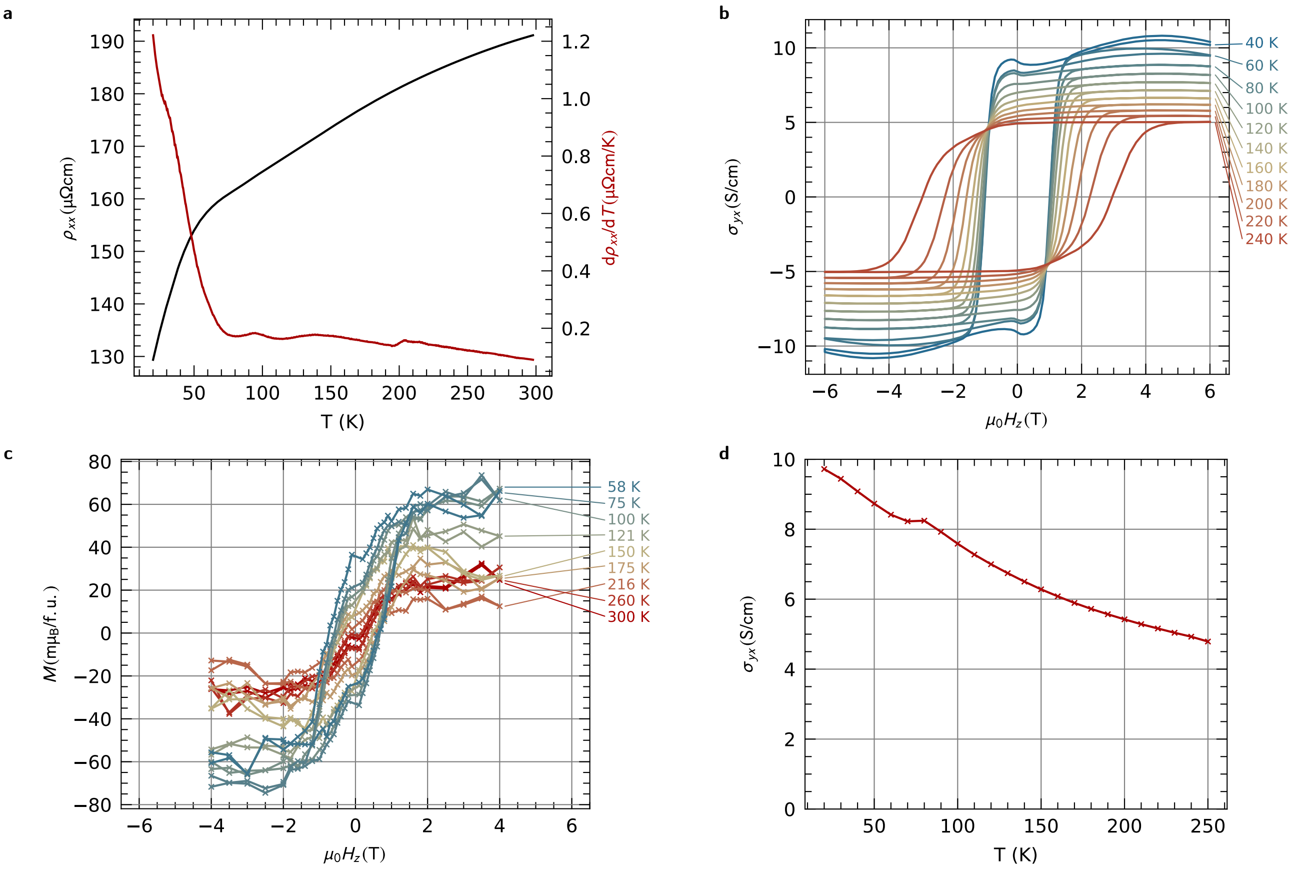

FIG. S1. Transport and magnetic characterization of Mn_5Si_3 samples. **a** Temperature dependence of resistivity and its first derivative. **b** Magnetic field dependence of the anomalous Hall contribution to transverse conductivity σ_{yx} for multiple temperatures between 40 K and 240 K. **c** Magnetic field dependence of magnetic moment detected by SQUID magnetometry for multiple temperatures between 58 K and 300 K. **d** Temperature dependence of the spontaneous (zero-field) anomalous Hall conductivity σ_{yx}.

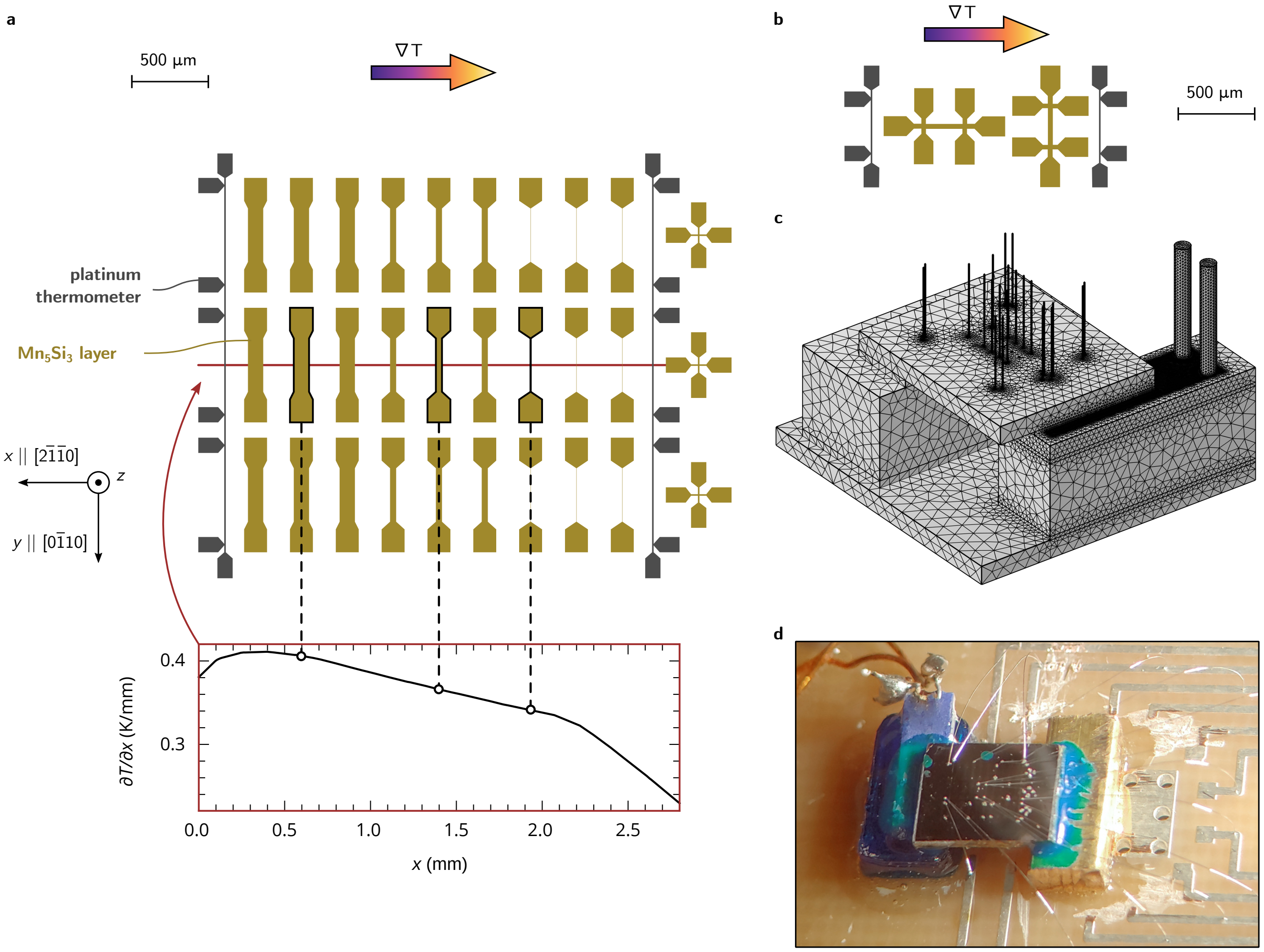

FIG. S2. The experimental configuration and lithographic design of Mn_5Si_3 samples. **a** The lithographic design used for the measurement of S_{yx}, including the simulated distribution of the in-plane temperature gradient at 121 K. The design consists of Mn_5Si_3 bars and devices prepared by ion-beam etching and sputtered platinum bars used as thermometers. The figure includes the spatial profile of the temperature gradient along the x-axis and on the sample surface, which was determined by finite-element modelling. The Nernst voltage was measured at four transverse bars marked `A`–`D`. The data represented in Fig. 1–4 correspond to the bar `B`. **b** The lithographic design used for the measurement of S_{xx}. The design consists of Mn_5Si_3 bars and devices and platinum bars used as thermometers. **c** The numerical mesh used for the finite-element simulation of the temperature distribution in our experimental setup. **d** A photographic picture of a Mn_5Si_3 sample in an experimental geometry for the measurement of the Nernst effect.

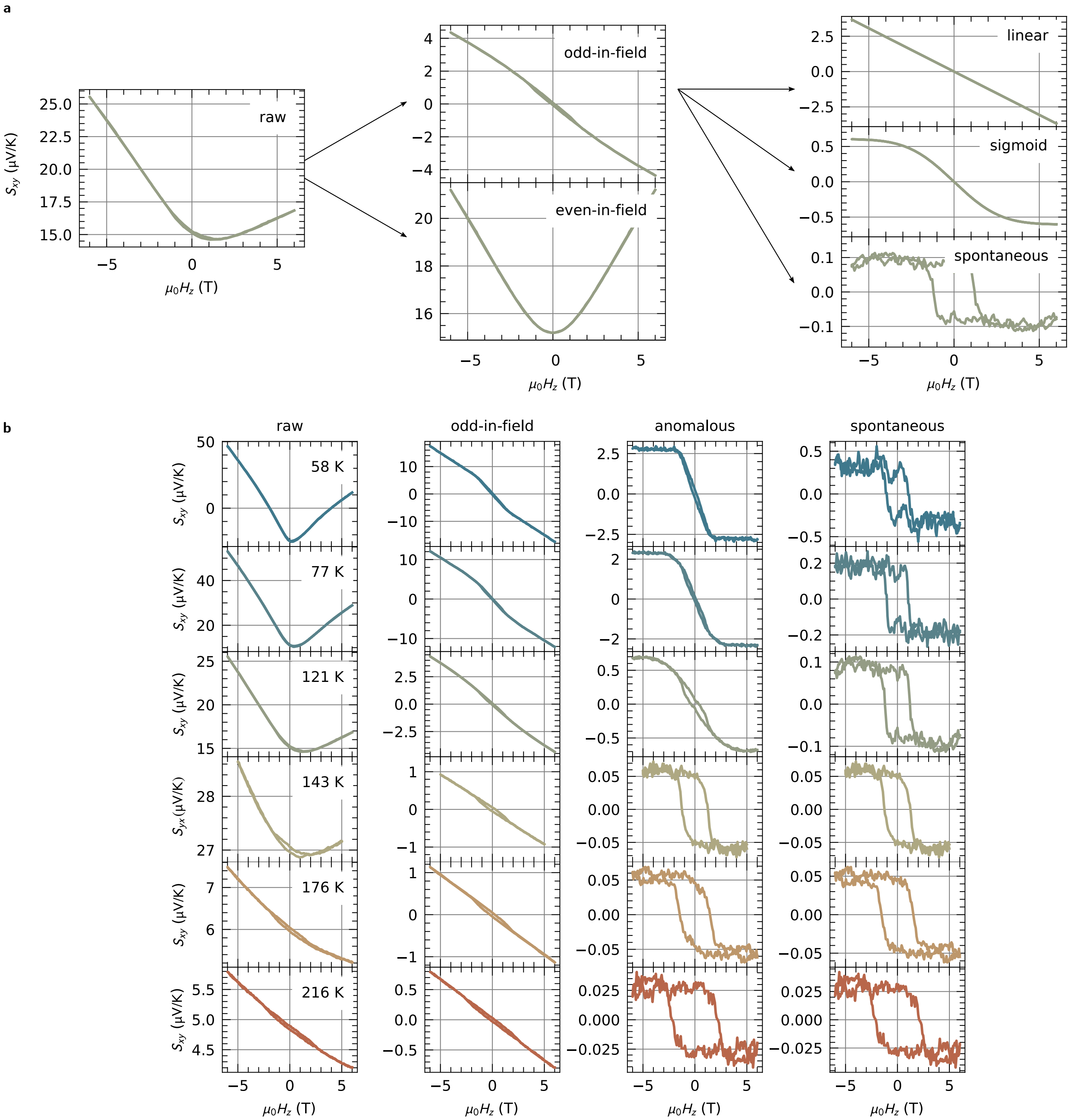

FIG. S3. Components of the S_{yx} signal. **a** We separate the raw field dependence of the transverse thermoelectric signal (left) to its even-in-field and odd-in-field components (centre). For the analysis of the Nernst signal, we take into account only the odd-in-field component. To the odd-in-field signal, we fit a linear and an error function (sigmoid) which allows us to separate the spontaneous contribution. The data correspond to the sample temperature of 121 K. **b** The field dependencies of raw, odd-in-field, anomalous (i.e. odd-in-field without the linear component), and spontaneous components of the S_{yx} signal measured at different temperatures.

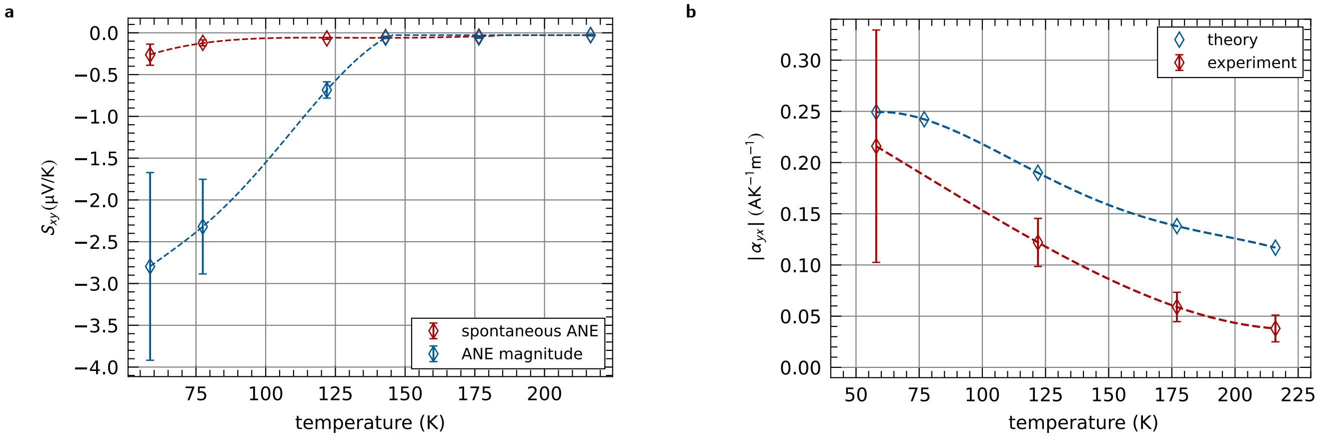

FIG. S4. **a** The temperature dependence of the ANE coefficient S_{xy} and its spontaneous ANE S_{xy} contribution. **b** The temperature dependence of the anomalous Nernst conductivity magnitude $| \alpha_{yx} |$ for calculated and measured values.

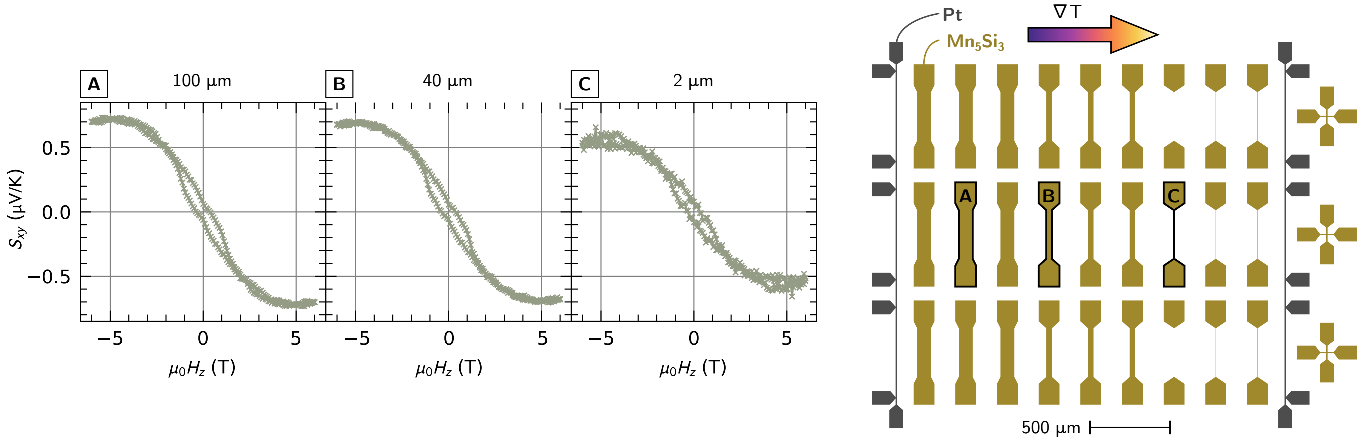

FIG. S5. Magnetic field dependence of anomalous Nernst coefficient S_{yx} measured at 121 K at multiple devices of our sample. The presented data labeled A-C correspond to the signals measured at the corresponding devices on the map. The width of devices' channel is 100 μm, 40 μm, and 2 μm respectively.

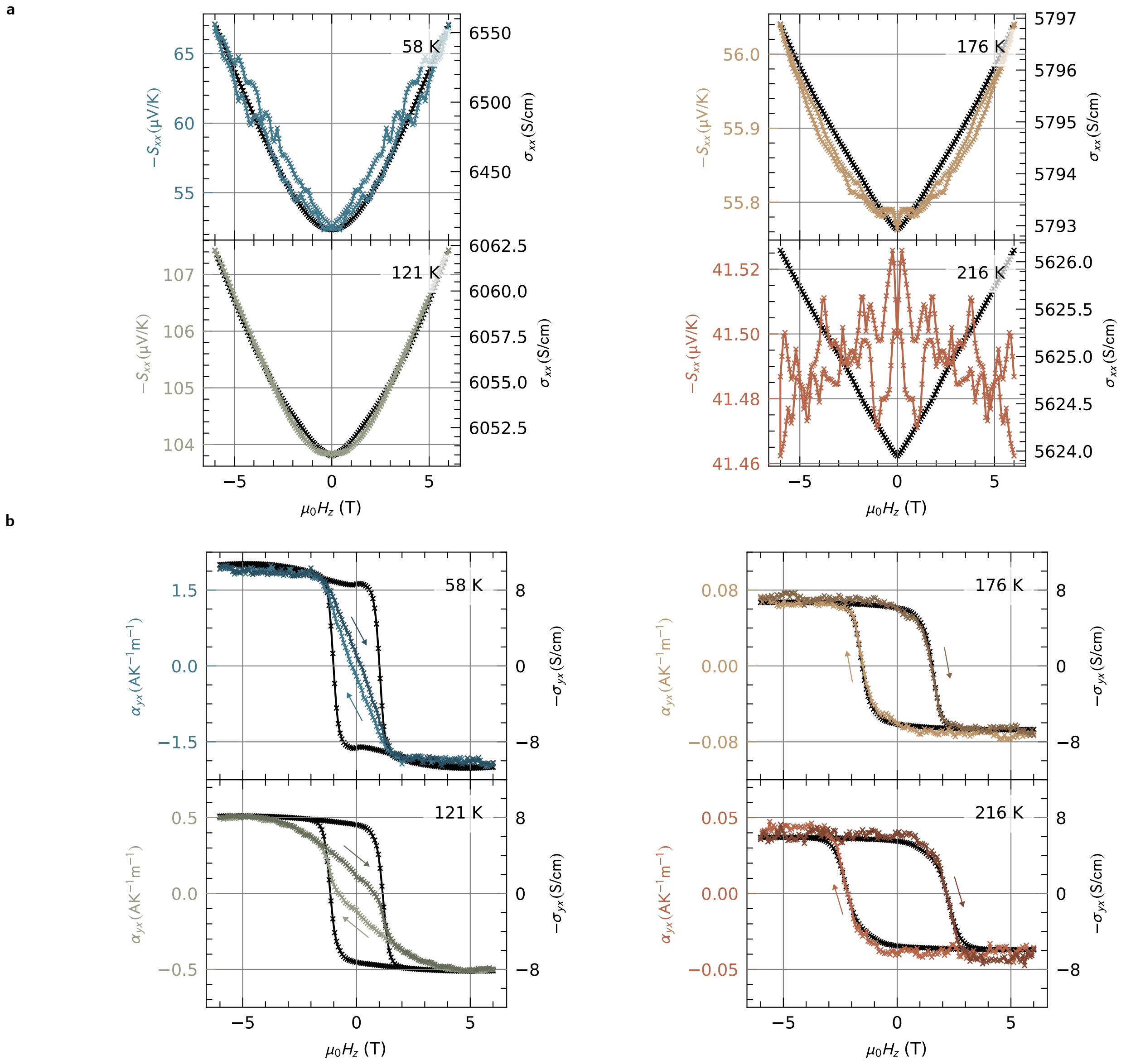

FIG. S6. Field dependence of coefficients and conductivities in Mn_5Si_3. **a** The field dependence of S_{xx} measured at different temperatures, including the field dependencies of σ_{xx} **b** The field dependence of the calculated α_{yx} at different temperatures, including σ_{yx} for comparison.

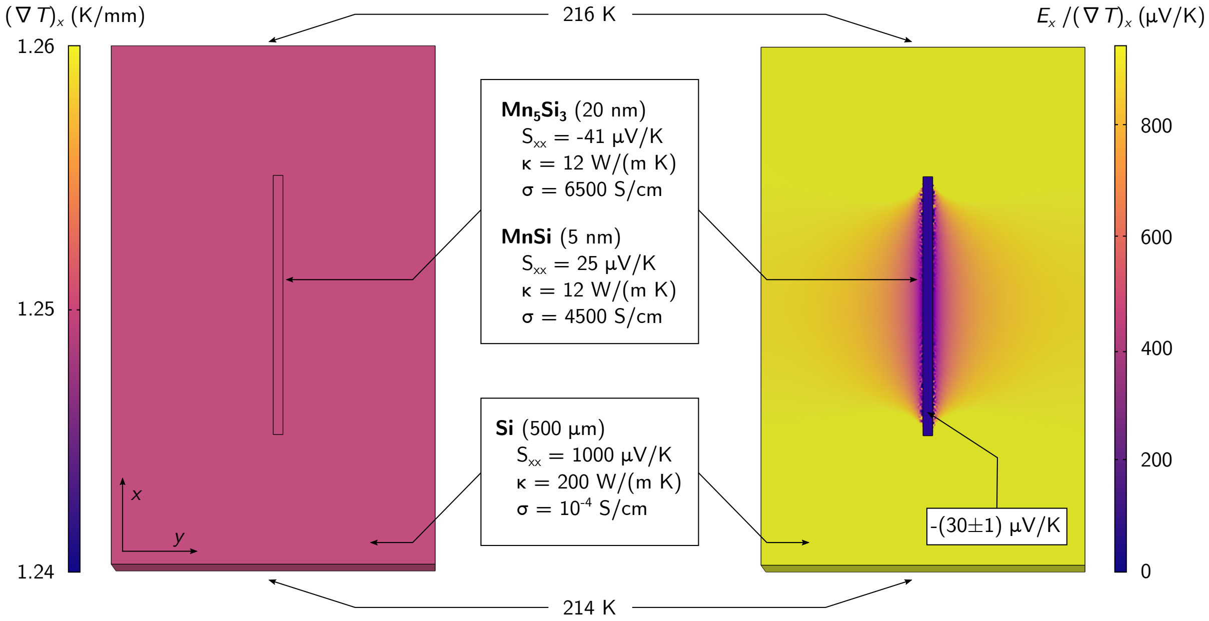

FIG. S7. Finite-element simulations of the Seebeck coefficient measurements at 215 K. The left panel shows the homogeneous distribution of the x-component of the temperature gradient at a device comprised of a Mn_5Si_3 / MnSi bilayer on a silicon substrate. The right panel shows the simulated Seebeck signal (i.e. longitudinal electric field over longitudinal temperature gradient) on the same device. The parameters in the center correspond to the used simulation values of the Seebeck coefficient, thermal conductivity, and electric conductivity. The simulated Seebeck signal is homogeneous at the device and reaches $-(30 \pm 1)\,\mu$V/K.

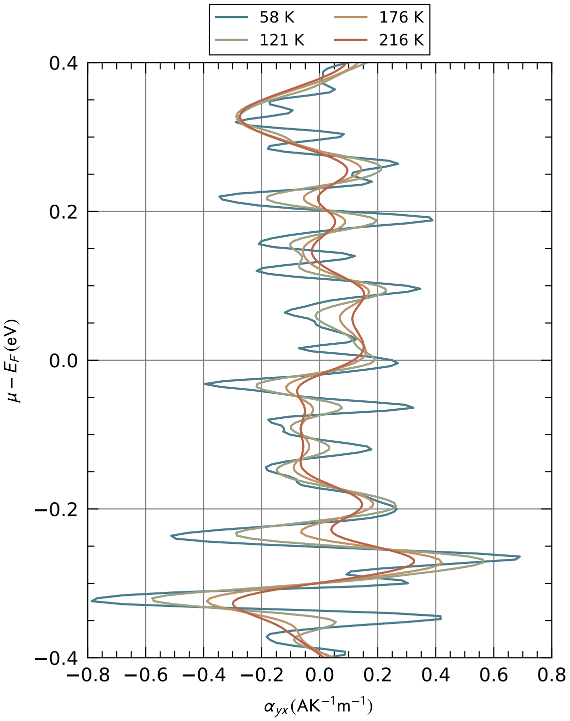

FIG. S8. The *ab initio* calculation of spontaneous Nernst conductivity in Mn_5Si_3. The figure features the calculated energy dependence of α_{yx} at different temperatures.

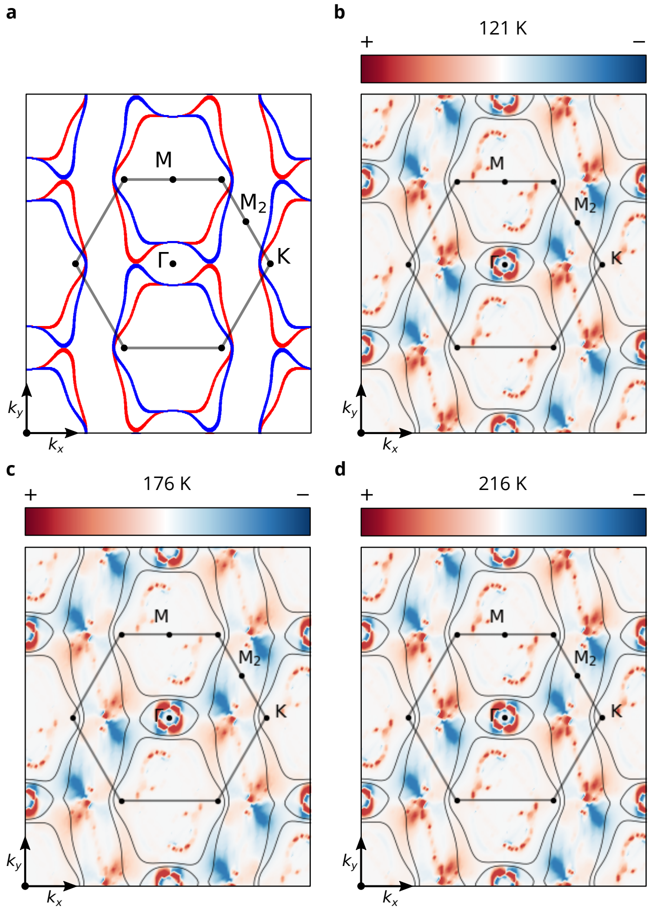

FIG. S9. Nonrelativistic Fermi surface and momentum-resolved distribution of the anomalous Nernst conductivity along the $k_z = 0$ plane. **a** Spin-resolved nonrelativistic Fermi surface with grey solid lines outlining the first Brillouin zone. The color map shows the spin texture of the Fermi surface, with spin-up (-down) energy bands in red (blue). **b-d** Distribution of the momentum-resolved anomalous Nernst conductivity for the temperatures 121 K, 176 K, and 216 K, respectively. The colormap (red (blue) for positive (negative) values) of each panel b-d was rescaled individually for improved visualization of the anomalous Nernst conductivity distribution over the Brillouin zone.

material	C_p (J kg^{-1}K^{-1})	κ (W K^{-1}m^{-1})
silicon	*[1]	*[2]
brass	380	40
ASA	1300	0.17
GE varnish	983	*[3]
aluminium	900	238
alumina ceramics	730	23

TABLE S1. Values of heat capacity at constant pressure C_p and thermal conductivity κ used for the finite-element modelling of the temperature gradient. For values marked by an asterisk, we used temperature-dependent C_p or κ.

[1] P. Flubacher, A. Leadbetter, and J. Morrison, The heat capacity of pure silicon and germanium and properties of their vibrational frequency spectra, Philosophical Magazine **4**, 273 (1959).
[2] C. J. Glassbrenner and G. A. Slack, Thermal conductivity of silicon and germanium from 3 K to the melting point, Physical review **134**, A1058 (1964).
[3] D. E. Tsatis, Thermal diffusivity of GE-7031 varnish, Journal of applied physics **62**, 302 (1987).
